# Analysis of the Expression and Mutation of Several Genes Related to Hearing Loss in Children in Vietnam

**DOI:** 10.7759/cureus.104119

**Published:** 2026-02-23

**Authors:** Phuong Thi Hoang, Nam Thanh Quan, Cuong Xuan Hoang, Thuy Thi Bich Vo

**Affiliations:** 1 Audiology and Vestibular Center, 108 Military Central Hospital, Hanoi, VNM; 2 Otolaryngology, 103 Military Hospital/Vietnam Military Medical University, Hanoi, VNM; 3 Internal Medicine, Vietnam Military Medical University, Hanoi, VNM; 4 Institute of Biology, Vietnam Academy of Science and Technology, Hanoi, VNM

**Keywords:** cochlear implantation, gene expression, hearing loss, mutation, vietnam

## Abstract

Background

Genetic mutations in *GJB2* and *GJB3* are primarily associated with non-syndromic sensorineural congenital hearing loss (HL), whereas mutations in *SLC26A4* and MT-RNR1 are linked to syndromic or mixed forms of sensorineural HL and are among the most commonly investigated hearing loss-associated genes. However, the clinical significance of these mutations, particularly in congenital HL, remains underexplored.

Methodology

In this study, 30 children with HL were evaluated for clinical features and tested for the expression of *GJB2*,* GJB3*,* SLC26A4,* and *MT-RNR1* using reverse transcription-polymerase chain reaction, with genetic analyses performed before cochlear implantation. Additionally, *MT-RNR1* was sequenced in all patients to detect potential mutations.

Results

Relative to *18S rRNA*, the expression levels of *GJB2, GJB3, SLC26A4*, and *MT-RNR1* were approximately 40%, 43.3%, 40%, and 46.67%, respectively. Six *MT-RNR1* variants were identified, including c.1438A>G, c.1119T>C, c.1048C>T, c.1107T>C, c.1541T>C, and c.1005T>C. While the m.1105T>C variant has been implicated in aminoglycoside-induced ototoxicity, no significant association was observed in this cohort.

Conclusions

These findings provide preliminary insights into the potential utility of gene expression and mutation screening for early diagnosis and risk assessment of congenital HL in Vietnam.

## Introduction

Hearing loss (HL) and deafness are major global health concerns and are broadly classified into sensorineural HL, which is most often associated with genetic causes, and conductive HL, which typically results from non-genetic abnormalities in sound transmission, affecting individuals of all ages and socioeconomic backgrounds. According to the World Health Organization (WHO), more than 1.5 billion people, about 20% of the global population, live with some degree of HL, and approximately 430 million suffer from disabling hearing impairment. Among these, an estimated 34 million are children, underscoring the global burden of pediatric hearing disorders [1]. In Vietnam, HL represents a growing public health issue. The country has one of the highest prevalence rates of HL in Southeast Asia, with more than 1.6 million people affected. Each year, between 1,200 and 1,400 infants are born with congenital deafness. The estimated incidence of congenital HL ranges from 1 in 500 to 3 in 1,000 live births. If left undiagnosed or untreated, HL in early life can have profound consequences on speech and language development, social interaction, psychological health, and academic achievement.

Congenital HL can result from both genetic and environmental factors. It is estimated that 50% of congenital HL cases are genetic in origin, while the remaining half result from environmental causes such as infections during pregnancy, ototoxic drugs, or perinatal complications [2]. Genetic HL can be syndromic or non-syndromic, with the latter accounting for approximately 70% of cases. Recent studies have identified over 600 gene loci associated with HL, and more than 150 genes have been confirmed to contribute to non-syndromic forms [3]. In Vietnam, several clinic-based studies have investigated deafness-associated genes, including *GJB2*, *SLC26A4*, *GJB3*, and *MT-RNR1*. Mutations in these genes can lead to HL at birth, during early childhood, or in response to specific environmental triggers. A well-established example of gene-environment interaction involves *MT-RNR1* mutations, in which carriers are highly susceptible to profound sensorineural HL following exposure to aminoglycoside antibiotics, even at standard therapeutic doses [4,5]. According to some recent studies worldwide, there are more than 200 mutated genes related to congenital HL [6], with a total of 18 common HL genes [7], four of which are *GJB2*, *SLC26A4*, *GJB3*, and *MT-RNR1* [3]. In addition to the genes located on the chromosomes in the nucleus, there are also genes located outside the mitochondria. The *MT-RNR1* gene encodes *12S rRNA*. Mutations in this gene cause mitochondrial ribosomes to become more similar to bacteria, increasing their susceptibility to aminoglycosides, antibiotics linked to damage to hair cells and the auditory nerve. Currently, few studies have evaluated the expression of these four similar genes in children with congenital HL, especially the role of point mutations in the *MT-RNR1* gene inherited from mothers of at-risk children. One of them increased the risk when aminoglycoside antibiotics were used. Therefore, this study aimed (i) to evaluate the expression levels of four genes (*GJB2*, *SLC26A4*, *GJB3*, and *MT-RNR1*) to support the diagnostic characterization of congenital HL; (ii) to screen for the *MT-RNR1* point mutations for early identification of congenital deafness; and (iii) to assess genetic susceptibility to aminoglycoside-induced HL, thereby informing risk stratification and preventive strategies in children exposed to aminoglycoside antibiotics.

## Materials and methods

A total of 30 children with congenital HL were recruited using a convenience sampling method, with peripheral blood samples collected between January and March 2025. The age of participants ranged from 12 to 60 months, and all children were under six years of age at the time of blood collection. All cases were diagnosed with congenital HL, with definitive diagnosis established before 36 months of age. Participants underwent cochlear implantation at Military Central Hospital 108 and several affiliated hospitals in Hanoi, Vietnam. The limited sample size reflects the availability of eligible pediatric patients during the study period and the high cost of cochlear implant devices.

Eligibility criteria included a confirmed clinical diagnosis of HL with successful cochlear implantation, completion of preoperative psychological and cognitive assessments, and availability of complete paraclinical data. Temporal bone CT and brain MRI revealed no cochlear malformations, with preserved cochlear structures and an intact vestibulocochlear nerve. Children were excluded if they did not meet these criteria, declined participation, or if biological samples were inadequately collected or stored.

Peripheral blood was selected as the biological sample because cochlear tissue is not ethically or clinically obtainable in children, and blood sampling is minimally invasive and suitable for genetic analyses. For each participant, 2 mL of peripheral venous blood was aseptically collected into EDTA-containing tubes, properly labeled, and handled to prevent contamination. Clinical examinations and audiological assessments were performed by otorhinolaryngology (ENT) specialists. Peripheral blood sampling was performed by trained pediatric nurses following standard clinical procedures. Pre-sampling counseling, explanation of the study objectives, and genetic counseling for parents or legal guardians were provided by physicians with expertise in otorhinolaryngology and/or medical genetics.

This study was approved by the Ethics Committee of the Institute of Genome Research (approval number: 01-2025/NCHG-HĐĐĐ, dated January 18, 2025). All procedures were conducted under the Declaration of Helsinki and national ethical guidelines.

RNA extraction and cDNA synthesis

Total RNA was extracted from blood with TRIzol Reagent (Invitrogen, Catalog Number 15596026) according to the manufacturer’s instructions [8]. This method allows efficient isolation of high-quality total RNA suitable for downstream gene expression analysis [9].

RNA concentration and purity were measured using the NanoDrop ND-1000 spectrophotometer (Thermo Scientific, USA) at 260/280 nm. cDNA synthesis was performed using the Tetro™ cDNA Synthesis Kit (Bioline, UK), following the supplier’s instructions [10].

Reverse transcription polymerase-chain reaction method

Gene-specific primers (Table 1) targeting *18S rRNA*, *GJB2*, *GJB3*, *SLC26A4*, and *MT-RNR1 *were used for endpoint reverse transcription polymerase-chain reaction (RT-PCR). Each PCR reaction was performed in duplicate (two technical replicates) for each sample, and no-template controls (NTCs) were included in every run to monitor potential contamination. The PCR reactions were performed using an Eppendorf Mastercycler Pro S (Eppendorf, Germany) under standard cycling conditions. PCR products were resolved on 1% agarose gels alongside a 100 bp DNA ladder and visualized using a GelDoc imaging system (Bio-Rad, USA). Primer specificity was confirmed by the presence of a single band corresponding to the expected amplicon size [11].

**Table 1:** Primer sequences of hearing loss genes and the housekeeping gene. Tm: melting temperature

Primer name		Seq (5’ -> 3’)	Size (bp)	Tm (°C)
*18S rRNA*	FW	AGCTCTTTCTCGATTCCGTG	110	57.7°C
	RV	GGGTAGACACAAGCTGAGCC		60.4°C
*GJB2*	FW	CTCACCGTCCTCTTCATTTT	531	59.4°C
	RV	ATTCCAGACACTGCAATCATGAAC		61.8°C
*SLC26A4*	FW	CTTACCAAGGAACAGTGTGTA	414	57.4°C
	RV	CCTGTTGCAATACTGGACAA		56.4°C
*GJB3*	FW	ACAACTACTTCCCCATCTCCAA	369	60.3°C
	RV	AGGTGAAGATTTTCTTCTCGGTA		59.3°C
*MT-RNR1*	FW	AAGCCGGCGTAAAGAGTGT	800	57.3°C
	RV	TGGTTTGGCTAAGGTTGTCTG		59.4°C

Relative gene expression was interpreted in reference to the internal control gene, *18S rRNA*, which was normalized to a value of 1. Expression values ≥0.85 were considered upregulated, values between 0.2 and 0.85 were considered unchanged, and values <0.2 were considered downregulated or absent.

Statistical analysis

The expression levels of genes were analyzed by measuring the brightness of the electrophoresis bands via Quantity One software (Bio-Rad, USA). The expression of each target gene was normalized to the level of the *18S rRNA* gene. Measurements were repeated for a minimum of two measurements. Consistency between duplicate reactions was assessed based on concordant band presence and intensity on agarose gels; no formal variability metrics were calculated due to the semi-quantitative nature of endpoint RT-PCR.

Gene sequencing and data analysis

Nucleotide sequences were determined by an ABI 3500 sequencer using specific primers for the *MT-RNR1* gene. The sequence of the sequenced gene was assembled with the reference sequence in GenBank (National Center for Biotechnology Information) and analyzed by BioEdit software to identify differences in the sequence of the replicated gene fragment.

## Results

Clinical and subclinical characteristics and gene expression levels

The clinical characteristics and screening results of the 30 patients whose deafness genes were tested are listed in Table 2.

**Table 2:** Clinical characteristics of 30 patients.

Characteristics	Number (%) of patients
Age (≤36 months)	100.00%
Sex	-
Male	16 (53.33%)
Female	14 (46.67%)
Premature birth	9 (30.00%)
Birth weight (<1.5 kg)	1 (3.33%)
Malformation diagnosis	8 (26.67%)
Have been diagnosed with a psychological problem	6 (20.00%)
Mother got sick during pregnancy	12 (40.00%)
Mother using drugs during pregnancy	1 (3.33%)
Jaundice increased bilirubin	4 (13.33%)
History of otitis media, encephalitis	2 (6.67%)
Other comorbidities	7 (23.33%)
Family history of hearing loss	4 (13.33%)

Descriptive analysis of the clinical characteristics of the patients (Table 2) showed that 14 of 30 participants were female (46.67%) and 16 of 30 were male (53.33%). This observed difference is descriptive only, and no inferential conclusions regarding sex-related prevalence were drawn due to the limited sample size. The HL time of all patients was under three years (≤36 months). Pediatric patients also indirectly experience several abnormal clinical features, such as maternal pregnancy status and possible delivery status; maternal disease during pregnancy; and an accompanying history, such as preterm birth, birth weight, and other pathological combinations. This may be related to congenital HL in children. Four samples (13.33%) had a family history of HL, which may be a genetic predisposition to congenital HL; however, the genetic issue of congenital deafness will be addressed in further studies.

**Figure 1:**
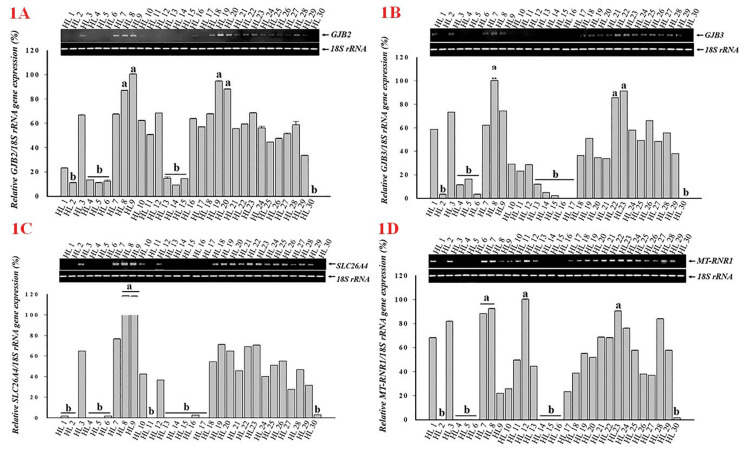
Relative expression levels of four target genes compared to the 18S rRNA reference gene in 30 children with hearing loss. (A)* GJB2* expression relative to *18S rRNA*. (B) *GJB3* expression relative to *18S rRNA*. (C) *SLC26A4* expression relative to *18S rRNA*. (D) *MT-RNR1* expression relative to *18S rRNA*. Gene expression ratios were interpreted as follows: values from 0.85 to 1 were considered upregulated (marked “a”); values <0.2 were considered downregulated (marked “b”).

The expression levels of the target genes *GJB2*, *GJB3*, *SLC26A4*, and *MT-RNR1* were evaluated by RT-PCR and compared to the housekeeping gene *18S rRNA* in 30 children with HL. Figure 1 illustrates the relative expression ratios of each gene across all patients, including indications of upregulation and downregulation (Figure 1).

Statistical results and analysis of the expression of all four genes, *GJB2*, *GJB3*, *SLC26A4*, and *MT-RNR1*, divided into two groups: (a) a group of patients with strong gene expression and another group (b) of patients with weak gene expression.

A single sample, HL8, presented increased expression of all four genes, *GJB2* (86.83%), *GJB3* (100%), *MT-RNR1* (92.15%), and *SLC26A4* (100.6%); however, when associated with clinical features, no differences were found in this patient. Some other patients have increased expression levels of one to two genes (increased expression of two genes, such as HL9 and HL23, or increased expression of one gene, such as HL12, HL19, HL20, and HL22), which are associated with specific clinical conditions. Specifically, abnormalities in the ear, such as bilateral inner ear malformations, bilateral vestibular drainage (HL9), or complications during the mother’s pregnancy (such as maternal fever of unknown origin, rubella infection, fetal distress, threatened miscarriage, and preterm delivery), can occur. In addition, the HL7 patients had only one *MT-RNR1* gene whose expression increased, but there was a family member with HL (Table 3).

**Table 3:** The expression of four target genes vs. the control gene (up and down two-fold change compared with 18S rRNA gene) and clinical features of 30 patients.

Samples	GJB2	GJB3	MT-RNR1	SLC26A4	CT and MRI findings	Clinical feature
HL 1	23.25	58.70	68.04	1.63	Normal temporal bone image on CT and MRI	Autism tracking. The mother had flu at <12 weeks pregnant
HL 2	10.92	3.35	0.00	0.00	Normal temporal bone image on CT and MRI	Being born prematurely <28 weeks. Jaundice, phototherapy, respiratory failure, mechanical ventilation >5 days. Congenital atrial septal defect, at 12 months examination found normal
HL 3	66.74	73.20	81.77	64.56	Normal temporal bone image on CT and MRI	Maternal pregnancy history and neonatal history were not unusual
HL 4	13.32	11.37	0.00	0.00	CT: Image of bilateral cochlear nerve stenosis; narrowing of the inner ear canals on both sides. MRI 1.5 tesla: Narrowing of the inner ear canal on both sides; bilateral cochlear nerve stenosis. No bilateral cochlear nerve was observed. MRI 3.0 tesla: Image of hypoplasia of the eighth nerve segment in the inner ear canal; no right cochlear branch was observed; observed left bundle branch streak/no abnormalities in brain parenchyma and cerebral vessels	Slight delay in motor and language development for age. Born prematurely at 32 weeks. Jaundice, increased bilirubin, 7 days of phototherapy
HL 5	11.03	16.19	0.00	0.00	CT: Image of narrowing of the left inner ear canal. Cochlear stenosis bilaterally. MRI: Narrowing of the left inner ear canal. Cochlear stenosis bilaterally. There are bilateral cochlear nerves. No other abnormalities of the skull or ears were found	Autism tracking -Jaundice, increased bilirubin, phototherapy for 7 days
HL 6	12.35	3.32	0.00	1.58	Normal temporal bone image on CT and MRI	Maternal pregnancy history and neonatal history are not unusual
HL 7	67.38	62.08	88.22	76.54	Normal temporal bone image on CT and MRI	The mother has risk factors for miscarriage -Uncle is hearing loss
HL 8	86.83	100.00	92.15	100.60	Normal temporal bone image on CT and MRI	Maternal pregnancy history and neonatal history are not unusual
HL 9	100.44	74.40	21.90	100.20	CT: Image of bilateral inner ear malformations: lower cochlea 2 and a half rings, small cochlea, short interseptum (type III according to Sennaroglu classification); underdeveloped semicircular canals. Widening vestibular drain on both sides. Narrow round windows on 2 sides. Small side stirrups. MRI: Image of bilateral inner ear malformations: The septum between the apical ring and the base of the cochlea is short on both sides. Underdevelopment of bilateral semicircular canals. Wide culverts on both sides. Narrowing of the inner ear canal. Bilateral cochlear nerve branches are also observed on MRI. Thickened left maxillary sinus mucosa, less left mastoid sinus fluid	Maternal pregnancy history and neonatal history are not unusual
HL 10	62.18	28.89	25.61	42.45	Normal temporal bone image on CT and MRI	Maternal pregnancy history and neonatal history are not unusual.
HL 11	50.50	22.96	49.53	0.00	CT: Soft tissue organization in the right outer ear and calcification in the left outer ear causing narrowing of the outer ear canal; need to coordinate otoscopy. MRI: Normal	Born prematurely at 38 weeks. Wanderbarg syndrome: hearing loss, blue eyes
HL 12	68.55	28.61	100.08	36.63	Normal temporal bone image on CT and MRI	Suspected autism. Born prematurely at 37 weeks
HL 13	14.64	11.96	44.48	0.00	CT: image of small bone cleft in the right upper semicircular canal. Chronic otitis media on the left, thickening of the lining of the left atrium, and the left mastoid may be less neutropenic. MRI: There are no abnormalities of cochlear nerve and inner ear structures on both sides on MRI. Thickening of bilateral maxillary sinus mucosa, left mastoid fluid	Mom has mumps. Have a family history of hearing loss. Inflammation of the middle ear
HL 14	9.32	4.70	0.00	0.00	Normal temporal bone image on CT and MRI	Mom has flu at 12 weeks pregnant
HL 15	14.46	2.18	0.00	0.00	Common compartment malformation. CT: Bilateral inner ear malformations: Type II incomplete fecal cochlear malformation, bilateral vestibular drain. Occipital and mastoid opacities (P). MRI: Image of inner ear malformations: Type II incompletely divided cochlear malformation, dilated ducts and bilateral fluid sacs. Auricular fluid collection and mastoid cyst (P)	The first sister, born in 2014, is congenital deafness, the patient is the 3rd child
HL 16	63.64	0.12	0.00	2.49	Normal temporal bone image on CT and MRI	Normal temporal bone image on CT and MRI
HL 17	56.89	0.14	23.29	0.00	Normal temporal bone image on CT and MRI	Normal temporal bone image on CT and MRI
HL 18	67.69	36.29	38.81	54.30	Normal temporal bone image on CT and MRI	Normal temporal bone image on CT and MRI
HL 19	94.64	50.84	55.06	71.12	Normal temporal bone image on CT and MRI	Rubella under <12 weeks pregnant
HL 20	88.02	34.38	51.74	64.74	Normal temporal bone image on CT and MRI	Unexplained high fever
HL 21	55.68	33.69	68.52	45.39	Normal temporal bone image on CT and MRI	
HL 22	59.30	85.39	68.08	68.94	Normal temporal bone image on CT and MRI	Jaundice, phototherapy for 7 days
HL 23	68.49	91.19	90.30	70.52	Normal temporal bone image on CT and MRI	Cesarean section, pregnancy failure. Born prematurely at 34 weeks. Light weight, 1,400g
HL 24	56.01	57.92	76.04	39.96	Normal temporal bone image on CT and MRI	Maternal pregnancy history and neonatal history are not unusual
HL 25	44.38	48.93	57.73	50.91	Normal temporal bone image on CT and MRI	Maternal pregnancy history and neonatal history are not unusual
HL 26	47.44	66.08	37.85	55.07	Normal temporal bone image on CT and MRI	Maternal pregnancy history and neonatal history are not unusual
HL 27	51.37	48.19	36.76	27.50	Normal temporal bone image on CT and MRI	Maternal pregnancy history and neonatal history are not unusual
HL 28	58.64	55.49	83.79	46.73	Normal temporal bone image on CT and MRI	Maternal pregnancy history and neonatal history are not unusual
HL 29	33.40	37.92	57.71	31.56	Normal temporal bone image on CT and MRI	Maternal pregnancy history and neonatal history are not unusual
HL 30	0.00	0.07	1.47	2.81	Normal temporal bone image on CT and MRI	Fairly intelligent, mildly active, a bit short-tempered, without anxiety and depression, able to integrate with friends. The mother had flu at 12 weeks pregnant

The gene expression level decreased evenly across four genes in eight samples (<20%), namely, HL2, HL4, HL5, HL6, HL14, HL15, and HL30. The common features of the patients were preterm birth, jaundice, hyperbilirubinemia, and structural abnormalities of the ear (H2, H4, H5, and H15). Patients H15 (with all four genes whose expression was reduced) and H13 (with 3/4 gene expression being reduced) all had factors related to the family of someone with HL. Although no ear abnormalities were detected in patients H14 and H30, the pregnant mothers were infected with influenza in the early stages of pregnancy. Patient H11, without *SLC26A4* gene expression, presented ear abnormalities and Wanderbarg syndrome (Table 3).

*MT-RNR1* gene mutations

The nucleotide sequence of a gene fragment 800 bp in length corresponding to the primer design size of the *MT-RNR1* gene in 30 patient samples was compared with the reference sequence of the *MT-RNR1* gene (NC_012920.1) (Table 4).

**Table 4:** Sequence mutations on the MT-RNR1 gene. (*): “+” indicates heterozygous mutation; “-” indicates homozygous mutation. If no data was available for the zygosity status, the cell is marked as NA.

Number of mutations	Reference sequence	Sequence change	Point change	Change	Heterozygous or homozygous (*)
30	A	G	1438	c.1438A>G	-
2	T	C	1119	c.1119T>C	-
1	C	T	1048	c.1048C>T	-
1	T	C	1107	c.1107T>C	-
2	T	C	1541	c.1541T>C	-
1	T	C	1005	c.1005T>C	-

Compared with the reference sequence, all 30 samples showed a homozygous A>G substitution at nucleotide position 1438 of the *MT-RNR1 *gene. At position 1119 in the HL10 and HL26 samples, the nucleotide T changed to C. Sample HL21 presented a homozygous mutation in which 1048 changed the nucleotide C to T and 1107 changed the nucleotide T to C. Samples HL4 and HL12 presented homozygous mutations at position 1541 T>C. In particular, in sample HL25, a homozygous mutation appeared at position 1005 T to C. This mutation is hypothesized to be associated with ototoxicity caused by the aminoglycoside group. Patient HL25 had a history of otitis media, and it is likely that when antibiotics from the aminoglycoside group were used to treat this disease, it can cause ototoxicity.

## Discussion

The gene expression levels of *GJB2*, *GJB3*, *SLC26A4*, and *MT-RNR1* were evaluated in 30 patients with HL in terms of clinical and subclinical characteristics. This study aimed to determine whether the expression patterns of these genes are associated with congenital HL and whether sequencing of *MT-RNR1* could identify disease-related variants. The results obtained for the expression levels of these four genes were compared with those of the control gene, which could be used for screening before being used for genetic analysis in patients. Our findings showed differential expression patterns relative to the control gene, while *MT-RNR1* sequencing did not reveal pathogenic mutations associated with congenital HL. The MT-RNR1 gene was simultaneously sequenced in 30 samples to search for mutations related to congenital HL. Patients with altered expression of these genes may suffer from non-syndromic HL, which is not associated with visible abnormalities or syndromic features. *GJB2* encodes connexin 26, critical for potassium recycling and cochlear cell signaling. Mutations disrupt gap junctions, leading to cochlear hair cell dysfunction and hearing impairment [12]. In this study, altered expression of *GJB2* may reflect underlying mutations, potentially affecting protein structure and function.

The process of regenerating potassium used by hair cells to generate action potentials in response to sound waves seems to be active. Mutations in the *GJB3* gene can cause autosomal dominant neuropathic deafness. This is because gap junctions open and close to regulate the flow of nutrients, charged atoms (ions), and other signaling molecules from cell to cell. This affects the growth and maturation of cells in the epidermis [13]. Mutations in this gene cause an asymptomatic form of HL called DFNB4. This form of HL may appear before a child learns to speak (prelanguage) or begin after a child learns to speak (after language learning). Most people with DFNB4 also have an abnormally large vestibular duct (enlarged vestibular aqueduct, or EVA). Here, patient code HL15 also had clinical features of bilateral vestibular dilatation and decreased *GJB3* gene expression, resulting in congenital deafness and requiring cochlear implantation. However, the mutation level of the gene in this patient was not evaluated.

Changes in *SLC26A4* gene expression impair or eliminate pendrin activity, upsetting the balance of ions in the inner ear. These changes presumably affect the development of structures in the inner ear, including the cochlea and vestibular aqueduct. Studies have shown that changes in ion concentration also lead to the loss of sensory cells in the inner ear needed for hearing [14]. The results also revealed that, compared with the other three genes, the *SLC26A4* gene presented the greatest change in expression (14/30 patients presented changes in gene expression, of which 4/30 patients presented decreased gene expression, 8/30 patients presented no gene expression, and 2/30 patients presented increased expression).

The *MT-RNR1* gene, a mitochondrial gene of approximately 954 bp without an exon-intron structure, encodes the 12S ribosomal RNA, which plays a critical role in mitochondrial protein synthesis required for oxidative phosphorylation. Mutations in this gene increase the risk of HL, especially in people taking prescription antibiotics called aminoglycosides. This antibiotic readily binds to aberrant 12S rRNA, impairing the ability of mitochondria to produce proteins required for oxidative phosphorylation. Researchers believe that this unintended effect of aminoglycosides could reduce the amount of ATP produced in the mitochondria, increase the production of harmful byproducts, and ultimately cause cells to self-destruct (through apoptosis) programmatically [15]. Among the patients whose expression of the *MT-RNR1* gene increased or decreased, some disease features, such as jaundice, increased bilirubin, premature birth or maternal disease during pregnancy, and a history of other comorbidities, were detected. In particular, there are patients with genetic factors in the family who have HL (such as H7, H15). Infections acquired by the mother during pregnancy, such as rubella, cytomegalovirus, toxoplasmosis, and herpes, or maternal use of aminoglycoside antibiotics during pregnancy can cause congenital deafness in children [16]. Premature birth is also one of the causes of deafness in children because, in these cases, some organs in children are not fully developed [17].

The *MT-RNR1* gene sequencing results did not show an apparent association between the detected variants and HL in this cohort; however, this observation should be interpreted with caution due to the limited sample size and the absence of a control group. The variant locus m.1438A>G (rsID: rs2001030) in *MT-RNR1* has been identified with a high frequency (>1%) in the general population and often in at least 10 individuals with normal hearing [4,18]. Another variant nucleotide position at 1119T>C (rs397515724) is not well conserved; it has no clinical significance despite extensive research on this gene in cases of HL around the world and has been identified with a similar frequency in HL patients and controls (~1-5%) [19-21]. This variant is a common polymorphism observed in phylogenetic studies and is considered a characteristic marker of certain mitochondrial haplogroups [19,20,22,23]. At position 1048C>T (rs111033323), this variant is not expected to be clinically significant, as it has been reported to be a common polymorphism in some ethnic populations based on evolutionary phylogenetic studies [20,23-25]. The mutation 1107T>C (rs2000974) is thought to be of no clinical significance because it is common and has a similar frequency in people with HL and controls [23]. This region of mitochondrial DNA is not evolutionarily conserved [23], and this variant is part of known polymorphisms associated with mitochondrial haplogroups [23,26]. The point 1005T>C (rs111033179) has been identified in 3/128 (2.3%) Chinese children with HL, but there is no evidence that these patients have a family history of HL [26]. In addition, its variant has been reported as a benign polymorphism on MitoMap and has also been identified in 7/2703 individuals in the Human Mitochondrial Genome Database. This variant is highly conserved, possibly related to aminoglycoside ototoxicity. Currently, it is considered a potential susceptibility site for aminoglycoside ototoxicity. Patient HL13 also had a history of otitis media and the use of aminoglycosides, but no mutation was found. Therefore, the variant m.1105T > C may not cause disease in this case. Although the 1541T>C mutation site is currently found in two samples of patients, this mutation is associated with high-grade serous ovarian cancer [27], and its role in HL has not yet been confirmed; however, further studies with large-scale samples are needed.

In this cohort, several perinatal risk factors associated with congenital or early-onset HL were observed, including preterm birth (30%), neonatal hyperbilirubinemia (13.33%), and very low birth weight (<1,500 g). These factors have been widely reported as risk contributors to congenital and early-onset HL in previous studies [28,29]. Although this study was not designed to assess gene-environment interactions quantitatively, these observations suggest a multifactorial etiology and should be interpreted descriptively.

Overall, no significant associations were observed between the expression patterns of* GJB2*,* GJB3*,* SLC26A4*, and *MT-RNR1* and maternal pregnancy history, family history of HL, or neonatal factors such as prematurity (all p > 0.05). However, a subset of patients showed concordant upregulation or downregulation across all four genes in the absence of clear environmental risk factors, suggesting a possible genetic contribution to a non-syndromic HL phenotype. In contrast, cases with largely unchanged gene expression more often presented with perinatal or environmental risk factors. These findings support a multifactorial etiology of congenital HL, although no definitive genotype-phenotype correlation could be established in this cohort.

Limitations of the study

This study has some limitations. The relatively small sample size (n = 30) may affect the statistical power and generalizability of the findings. The absence of a normal-hearing control group limits direct comparative analyses, and the use of endpoint RT-PCR provides semi-quantitative rather than absolute gene expression measurements. Additionally, although *MT-RNR1* gene sequencing was performed, other potentially relevant genetic loci were not investigated. Nevertheless, the study provides important preliminary data that can inform future large-scale genetic studies in pediatric HL.

## Conclusions

Taken together, the gene expression findings provide valuable insights into the roles of *GJB2*, *GJB3*, *SLC26A4*, and *MT-RNR1 *in children with HL who have undergone cochlear implantation. Although six *MT-RNR1* variants were identified, no significant association with HL was observed, likely due to the limited sample size. Early detection of gene expression changes and pathogenic variants may potentially aid in the prevention of congenital HL, particularly in high-risk cases; however, prospective validation in larger cohorts is required before clinical implementation. Furthermore, timely diagnosis can support early intervention, including treatment planning, language development, and genetic counseling for patients and their families, and could be integrated as an adjunct to newborn hearing screening programs in Vietnam in the future, pending further validation.
